# Microbiota Characterization and Bioactive Potential of Broccoli (*Brassica oleracea* var. *italica*) By-Products: Implications for Sustainable Antimicrobial Applications

**DOI:** 10.3390/foods15101786

**Published:** 2026-05-18

**Authors:** Iris Gudiño, María José Benito, Alberto Martín, Rocío Casquete

**Affiliations:** 1 Nutrición y Bromatología, Escuela de Ingenierías Agrarias, Universidad de Extremadura, Avd. Adolfo Suárez s/n, 06007 Badajoz, Spain; igudino@unex.es (I.G.); amartin@unex.es (A.M.); rociocp@unex.es (R.C.); 2 Instituto Universitario de Investigación en Recursos Agrarios (INURA), Universidad de Extremadura, Avd. de la Investigación, 06006 Badajoz, Spain

**Keywords:** broccoli by-products, plant-associated microbiota, antimicrobial activity, glucosinolates, sustainable valorization

## Abstract

Broccoli (*Brassica oleracea* var. *italica*) by-products represent an abundant and underutilized source of bioactive compounds with potential applications in sustainable food systems. This study aimed to characterize the microbiota associated with different plant fractions (leaves, stems, and heads) of broccoli (Parthenon and Tritón cultivars) and to evaluate the antioxidant and antimicrobial properties of their extracts, using cauliflower as a reference. Microbial counts and fungal identification (ITS sequencing) were performed, while phytochemical profiles were analyzed by HPLC-ESI-QTOF. Antioxidant activity was assessed using DPPH and ABTS assays, and antimicrobial activity under in vitro conditions was evaluated against selected foodborne bacteria and phytopathogenic fungi. Broccoli by-products, particularly leaves, showed lower microbial loads in certain cultivars and were rich in phenolic compounds and glucosinolates; however, higher phenolic content did not always correlate with greater antioxidant activity, highlighting the importance of compound composition. All extracts showed strong antibacterial activity at higher concentrations, especially against *Listeria* spp. Notably, antifungal activity was selective but relevant, with consistent inhibition observed against *Alternaria alternata*, while *Penicillium purpurogenum* and *Botrytis cinerea* exhibited higher resistance. Overall, these findings highlight the potential of broccoli by-products as sustainable sources of natural bioactive compounds for food applications, particularly in the development of preservation strategies and postharvest treatments. Further studies focusing on individual compounds and their specific biological activities are needed to better understand the mechanisms underlying these effects and to support their application in real food systems.

## 1. Introduction

Broccoli (*Brassica oleracea* var. *italica*) is a widely consumed cruciferous vegetable with a strong presence in the global diet. It is highly valued for its recognized nutritional quality and health-promoting properties. Together with other *Brassica* vegetables, such as cauliflower (*Brassica oleracea* var. *botrytis*), broccoli constitutes an important horticultural crop worldwide, characterized by high production volumes and intensive cultivation systems.

The horticultural industry generates substantial amounts of by-products throughout the production chain, particularly during harvesting, handling, and processing stages. These by-products include plant materials that do not meet commercial standards, field residues, and fractions not intended for fresh consumption. In the case of broccoli, only the inflorescence or head is typically commercialized, while the remaining plant tissues—mainly leaves and stems—are considered by-products or waste. These fractions may account for 60–75% of the total plant biomass, with approximately 73.65 t/ha of plant residues generated after harvest [1]. Currently, these residues are largely reincorporated into the soil or used as animal feed. However, their valorization is of significant economic and environmental interest, as it enables the recovery of underutilized biomass while reducing the environmental impact associated with waste management.

Broccoli by-products represent a rich and diverse source of bioactive compounds, including glucosinolates, phenolic compounds such as flavonoids, dietary fiber, vitamins, and essential minerals. Notably, many of these compounds may be present at similar or even higher concentrations than those found in the edible inflorescence [1]. Numerous studies have highlighted the potential of broccoli by-products as sources of bioactive compounds for industrial applications, including the enhancement of the nutritional profile of foods [2], their use as natural alternatives to conventional additives [3], and their application as natural preservatives capable of extending food shelf life [4]. These phytochemicals have been associated with a wide range of biological activities, including antioxidant [5], anticancer [6], antimicrobial [7], and antifungal effects [8], reinforcing their potential as functional raw materials. However, the presence and concentration of these compounds are influenced by several factors, including cultivar, climatic conditions, plant developmental stage, agricultural practices (e.g., fertilization and pesticide use), and the specific plant fraction considered (inflorescence, leaves, or stems), resulting in considerable variability in the phytochemical profile [9].

The plant-associated microbiota plays a crucial role in crop health and represents a key factor in the microbial safety of plant-derived foods. Microbial contamination has been linked to multiple environmental and agronomic factors, including irrigation water quality, soil conditions, wildlife interactions, and inadequate agricultural practices [10,11]. In this context, the integration of good agricultural practices with the use of endogenous bioactive compounds represents a promising strategy to reduce contamination, minimize postharvest losses, and decrease reliance on synthetic chemical treatments. The year-round demand for broccoli necessitates extended storage under low-temperature and high-humidity conditions, which, together with environmental factors during cultivation, can promote the development of fungal diseases and contribute to postharvest losses [12,13]. Given the large volume of broccoli by-products generated and their richness in compounds with antifungal activity, these materials represent a sustainable alternative for developing pre- and postharvest treatments.

Glucosinolates and their hydrolysis products have demonstrated effectiveness against a broad spectrum of microorganisms, including bacteria and filamentous fungi, through mechanisms that disrupt cell integrity and interfere with essential metabolic processes [14]. Similarly, glucosinolate-rich leaf extracts have shown strong in vitro antifungal activity against several plant pathogens, such as *Alternaria*, *Sclerotinia*, and *Rhizoctonia*, particularly when combined with the enzyme myrosinase [15]. In addition, other broccoli-derived compounds and extracts have exhibited antimicrobial and antifungal activity against a range of microorganisms, including seed-derived proteins effective against *Fusarium culmorum* and *Penicillium expansum* [16]; stem and floret extracts active against pathogenic bacteria, phytopathogenic fungi (e.g., *Colletotrichum gloeosporioides*, *Aspergillus niger*), and yeasts [8]; and leaf extracts effective as postharvest antifungal treatments capable of extending the shelf life of horticultural products and inhibiting food-related microorganisms [7,17].

Previous studies reported have extensively characterized the phytochemical composition and biological activity of broccoli by-products, highlighting their potential as sources of bioactive compounds. However, these studies have primarily focused on chemical profiling and functional properties, without considering the role of plant-associated microbiota and its potential interaction with phytochemical composition and biological activity.

In this context, the present study aims to provide an integrated evaluation of broccoli by-products by combining microbiota characterization, phytochemical profiling, and bioactivity assessment across different plant fractions and cultivars. Unlike previous studies mainly focused on phytochemical composition or biological activity separately, this approach enables a more comprehensive understanding of the relationships between microbial load, chemical composition, and functional properties under in vitro conditions. This integrated perspective contributes to a more realistic evaluation of the potential of broccoli by-products for food-related applications and sustainable valorization strategies.

## 2. Materials and Methods

### 2.1. Plant Materials

For this study, cauliflower (*Brassica oleracea* var. *botrytis*) and broccoli (*Brassica oleracea* var. *italica*) plants were analyzed. Broccoli samples corresponded to two commercial cultivars, Parthenon and Tritón. All plant material was supplied by the company Delicias de Guadiana, whose cultivation fields are located in Extremadura (Spain; 39.0252° N, 5.9085° W). Approximately 1 kg of broccoli by-products, including leaves, stems, and heads, was obtained from Delicias del Guadiana (Extremadura, Spain). The plant material was collected from different production batches and pooled to obtain representative samples of each plant fraction and cultivar. Prior to analysis, samples were thoroughly homogenized using a laboratory grinder to ensure uniformity. For each plant fraction and cultivar, three independent biological replicates (corresponding to different batches) were processed, and all analytical determinations were performed in triplicate as technical replicates.

### 2.2. Bacterial and Fungal Strains

Four bacterial strains obtained from the Spanish Type Culture Collection (CECT) were used: *Bacillus cereus* CECT 131, *Staphylococcus aureus* CECT 976, *Listeria innocua* CECT 910, and *Listeria monocytogenes* CECT 911. In addition, a collection of fungal isolates obtained from broccoli and cauliflower by-products was included. These isolates were recovered from potato dextrose agar (PDA) plates inoculated with plant material (leaves, stems, and heads), as described in Section 2.3.

### 2.3. Microbial Counts and Isolation of Molds

Microbial counts were performed on the three plant fractions (leaves, stems, and heads) from cauliflower and from the two broccoli cultivars (Parthenon and Tritón). Samples were serially diluted in peptone water and plated on the culture media listed in Table 1.

Plates were incubated at the appropriate temperature for each microbial group for 24–48 h, and results were expressed as log_10_ CFU/g. All determinations were performed in triplicate.

Mold isolation was carried out based on colony morphology observed on acidified potato dextrose agar (PDA) plates (Condalab, Torrejón de Ardoz, Spain), adjusted to pH 3.5 using a sterile 10% (*w*/*v*) tartaric acid solution. Two to three representative colonies of each morphological type were selected from the highest dilution plates and subcultured twice on acidified PDA to obtain pure cultures. The purified isolates were subsequently stored in sterile glycerol at −80 °C.

### 2.4. Molecular Identification of Fungal Isolates

Genomic DNA from fungal isolates was extracted by direct cell lysis using the Quick-DNA Fungal/Bacterial Miniprep Kit (Zymo Research, Tustin, CA, USA), in accordance with the manufacturer’s instructions. Species identification was performed by amplification of the ITS1–ITS2/5.8S rRNA internal transcribed spacer region using primers ITS1 and ITS4 [13].

PCR amplification was carried out in a T100™ thermal cycler (Bio-Rad, Hercules, CA, USA) under the following conditions: initial denaturation at 95 °C for 3 min; 35 cycles of denaturation at 95 °C for 1 min, annealing at 52 °C for 1 min, and extension at 72 °C for 90 s; followed by a final extension at 72 °C for 10 min.

PCR products were purified using the GeneJET PCR Purification Kit (Thermo Fisher Scientific, Waltham, MA, USA) or the NucleoSpin Gel and PCR purification kit and subsequently sequenced at the Applied Biosciences Technical Service (STAB) of the University of Extremadura (Badajoz, Spain). Sequences were edited using Chromas Pro version 1.49 beta (Technelysium, Brisbane, Australia) and compared against EMBL/GenBank databases using the BLAST algorithm v2.17 available through the NCBI web server (National Center for Biotechnology Information, Bethesda, MD, USA). Identification was assigned based on the highest sequence similarity. Accession numbers of reference strains are provided in Section 3: Results.

### 2.5. Extraction of Bioactive Compounds

Bioactive compounds, particularly glucosinolates, were extracted following the method described by Tian et al. [18], with minor modifications. Briefly, 5 g of plant material was extracted with 15 mL of 70% (*v*/*v*) methanol using an ultrasonic bath for 20 min at 75–80 °C to inactivate myrosinase. Solid residues were removed by centrifugation (15,000 rpm, 15 min), and the extraction was repeated twice.

Methanol was removed under reduced pressure at 37 °C using a rotary evaporator. The resulting aqueous extracts were pooled and freeze-dried (Telstar, LyoBeta, Terrassa, Spain) for further analyses.

### 2.6. Determination of Total Phenolic and Flavonoid Contents

Total phenolic content was determined using the Folin–Ciocalteu colorimetric method [19]. Briefly, 0.01 g of extract was resuspended in 1 mL of absolute ethanol. An aliquot of 500 µL was mixed with 10 mL of Milli-Q water, 1 mL of Folin–Ciocalteu reagent, and 2 mL of 20% Na_2_CO_3_ solution, and diluted to a final volume of 25 mL. The mixture was incubated in the dark at room temperature for 1 h. Absorbance was measured at 760 nm, and results were expressed as mg gallic acid equivalents (GAE) per 100 g of dry extract.

Flavonoid content was determined following the method of Shraim et al. [20]. Briefly, 1 mL of sample was mixed with 3 mL of methanol, 0.2 mL of aluminum chloride (10% *w*/*v*), and 0.2 mL of potassium acetate (1 M). Subsequently, 5.6 mL of distilled water was added, and the mixture was incubated in the dark for 30 min. Absorbance was measured at 415 nm, and results were expressed as mg/L using a quercetin calibration curve.

### 2.7. Antioxidant Activity Assays

The antioxidant capacity of broccoli by-product extracts was evaluated using DPPH and ABTS radical scavenging assays, following the methods described by Teixeira et al. [21] and Cano et al. [22], respectively. Extracts (0.01 g) were dissolved in 1 mL of absolute ethanol prior to analysis. All assays were performed in triplicate, and results were expressed as mg Trolox equivalents per 100 g of dry weight.

### 2.8. Identification of Phenolic Compounds by HPLC-ESI-MS/MS

Phenolic compounds were analyzed using high-performance liquid chromatography coupled to electrospray ionization quadrupole time-of-flight mass spectrometry (HPLC-ESI-QTOF). Extract samples (0.08 g) were dissolved in 2 mL of HPLC-grade methanol, filtered, and subjected to chromatographic analysis using an Agilent 1260 Infinity HPLC system coupled to a quadrupole time-of-flight mass spectrometer (Model G6530, Agilent Technologies, Santa Clara, CA, USA).

Mass spectrometric detection was performed using an electrospray ionization (ESI) source operating in negative ion mode. Data acquisition was carried out over a mass-to-charge (*m*/*z*) range of 100–1700. The ion source parameters were set as follows: gas temperature, 280 °C; drying gas flow, 11 L/min; and nebulizer pressure, 35 psi.

Chromatographic separation was achieved using a gradient elution system consisting of water with 0.1% (*v*/*v*) formic acid (solvent A) and methanol with 0.1% (*v*/*v*) formic acid (solvent B), at a constant flow rate of 0.35 mL/min. The gradient program was as follows: initial conditions at 5% B, linearly increased to 90% B over 15–20 min, followed by re-equilibration to initial conditions over 10 min.

Compounds were tentatively identified using the MassBank database [23] and by comparison with previously reported data [5,24,25,26].

### 2.9. Antibacterial Activity

Antibacterial activity was evaluated under in vitro conditions against the bacterial strains described in Section 2.2, following the methodology previously described by Gudiño et al. [5], with minor modifications. Extracts were initially dissolved in dimethyl sulfoxide (DMSO) at a concentration of 10 mg/mL and subsequently diluted to final concentrations of 80, 60, and 40 ppm.

Bacterial suspensions were prepared from overnight cultures grown on brain–heart infusion (BHI) agar at 37 °C. Colonies were resuspended in sterile peptone water to achieve a turbidity equivalent to 0.5 McFarland. In sterile microplates, 2% (*v*/*v*) of each bacterial suspension was inoculated, followed by the addition of extract solutions. DMSO without extract served as a negative control.

All assays were performed in triplicate and incubated at 37 °C for 24 h. Microbial growth was monitored by measuring optical density using a FLUOstar Optima plate reader (BMG Labtech, Ortenberg, Germany). Results were expressed as percentage inhibition.

### 2.10. Antifungal Activity

Antifungal activity was assessed under in vitro conditions by measuring fungal growth on PDA plates, following the methodology described by Thery et al. [16], with modifications. The selected fungi (*Botrytis cinerea*, *Alternaria alternata*, and *Penicillium purpurogenum*) were previously isolated and identified as described in Section 2.3 and Section 2.4.

Spore suspensions (10^6^ spores/mL) were prepared from PDA cultures and quantified using a Neubauer chamber. Extracts (50 µL) at concentrations of 2500 and 1250 ppm were spread onto PDA plates, followed by inoculation with 2.5 µL of spore suspension.

Plates without extract served as positive controls, while water was used as a negative control. Incubation was carried out at 25 °C for 7 days. Fungal growth was measured in both radial and vertical directions, and results were expressed as percentage inhibition.

### 2.11. Statistical Analysis

Statistical analyses were performed using SPSS software (version 22.0, SPSS Inc., Chicago, IL, USA). One-way ANOVA was applied to physical variables influenced by a single factor, whereas two-way ANOVA was used for antimicrobial data to evaluate the interaction between extract concentration and sample type. When significant interactions were detected (*p* < 0.05), Tukey’s post hoc test was applied for multiple comparisons.

Principal component analysis (PCA) was performed to explore multivariate relationships among sample type, phytochemical composition, and the measured biological response variables.

## 3. Results

### 3.1. Microbial Characterization of Cauliflower and Broccoli Samples

The microbial counts obtained for cauliflower and broccoli (Parthenon and Tritón cultivars) are presented in Table 2, showing significant differences depending on both the plant fraction and the vegetable type.

Overall, cauliflower samples exhibited the highest microbial loads across most of the analyzed microbial groups, whereas broccoli, particularly the Parthenon cultivar, showed comparatively lower counts. This trend was consistent across molds, yeasts, mesophilic aerobic bacteria, and *Enterobacteriaceae*. These differences may be attributed to variations in plant morphology, tissue composition, and phytochemical profile. Cauliflower curds present a more compact and humid microenvironment that can favor microbial retention and growth, while broccoli tissues, especially in certain cultivars, are characterized by higher levels of bioactive compounds such as glucosinolates and phenolic compounds, which may exert inhibitory effects on microbial proliferation. In addition, cultivar-dependent factors, including structural characteristics and metabolic composition, may further contribute to the reduced microbial loads observed in the Parthenon samples.

Mold counts were significantly higher in cauliflower by-products, especially in stems (5.42 log CFU/g) and leaves (4.86 log CFU/g), compared to broccoli samples, where values ranged from non-detectable levels to 2.74 log CFU/g. Notably, molds were not detected in Parthenon heads, suggesting a reduced susceptibility of this fraction or lower exposure to environmental contamination sources.

In contrast, yeast counts were highest in cauliflower heads (4.78 log CFU/g), significantly exceeding those observed in broccoli samples, where yeasts were absent in most Tritón fractions and in Parthenon heads. This pattern may be related to differences in tissue composition and nutrient availability, which can favor yeast proliferation in reproductive structures such as the heads.

Mesophilic aerobic bacteria were abundant in all samples, with values ranging from 4.91 to 6.99 log CFU/g. The highest counts were recorded in cauliflower leaves (6.99 log CFU/g), while significantly lower levels were observed in broccoli stems, particularly in Parthenon (5.05 log CFU/g) and Tritón (4.91 log CFU/g). Similarly, Enterobacteriaceae counts were higher in cauliflower (up to 6.85 log CFU/g in leaves) than in broccoli, where the lowest levels were detected in Tritón stems (3.65 log CFU/g). These results suggest a greater degree of environmental contamination or less effective natural microbial inhibition in cauliflower.

Regarding specific microbial groups, *Staphylococcus aureus* and *Staphylococcus* spp. were detected in most samples, with higher counts generally observed in cauliflower and Tritón leaves. In contrast, their presence was limited or absent in broccoli heads, particularly in the Parthenon cultivar. Micrococcaceae counts showed less variability among samples, although higher values were observed in Tritón leaves and cauliflower heads.

Of particular concern is the detection of *Listeria monocytogenes*, which was present in several samples, notably in Tritón leaves (3.59 log CFU/g) and cauliflower stems (2.82 log CFU/g), while it was not detected in any of the Parthenon samples. This finding highlights potential food safety risks associated with certain plant fractions and cultivars.

Overall, the results indicate that microbial distribution is strongly influenced by plant fraction, with leaves generally showing higher contamination levels due to their greater exposure to environmental factors such as soil, irrigation water, and air. In addition, the observed differences between cauliflower and broccoli, as well as between broccoli cultivars, suggest that intrinsic plant characteristics, including the presence of bioactive compounds such as glucosinolates, may contribute to shaping the associated microbiota.

These findings reinforce the importance of considering both plant fraction and cultivar when assessing microbial safety and support the potential use of broccoli by-products, particularly from the Parthenon cultivar, as a lower-risk and functionally valuable raw material.

The molecular identification of fungal isolates obtained from cauliflower and broccoli by-products is presented in Table 3. Sequencing of the ITS region allowed reliable taxonomic assignment at the species level in most cases, with identity values ranging from 94% to 100%.

Overall, the fungal community was dominated by genera commonly associated with plant materials and postharvest environments, particularly *Alternaria*, *Penicillium*, and *Fusarium*. These genera are widely reported as ubiquitous colonizers of fresh produce and are frequently linked to spoilage processes and, in some cases, to mycotoxin production.

In cauliflower samples, the fungal population was mainly represented by *Alternaria alternata* and *Penicillium* species, including *P. purpurogenum* and *P. variabile*. The repeated identification of *A. alternata* across different isolates suggests its strong adaptation to cauliflower tissues, likely due to its ability to colonize senescent plant material and its well-documented role as a phytopathogen. Similarly, the presence of *Penicillium* species is consistent with their ecological role as saprophytic fungi capable of proliferating under postharvest conditions.

In the Parthenon broccoli cultivar, the fungal diversity was comparatively lower, with isolates identified as *Fusarium oxysporum* and *Penicillium* species (*P. dimorphosporum* and *P. purpurogenum*). The detection of *F. oxysporum*, a well-known soil-borne pathogen, suggests possible contamination originating from soil or irrigation water. However, the reduced diversity observed in this cultivar is in agreement with the lower microbial counts previously reported (Table 2), supporting the hypothesis that intrinsic plant factors, such as the presence of antimicrobial phytochemicals, may limit fungal colonization.

In contrast, the Tritón cultivar exhibited the highest fungal diversity among the analyzed samples. Several *Penicillium* species were identified, including *P. chalabudae*, *P. menonorum*, and *P. pimiteouiense*, along with *Alternaria alternata*, *Fusarium oxysporum*, and *Scolecobasidium ramosum*. This broader diversity may reflect differences in cultivar susceptibility, environmental exposure, or postharvest handling conditions. Notably, the identification of *S. ramosum* showed a lower similarity value (94%), which may indicate either a less well-characterized species or potential limitations in database coverage for this genus.

From a food safety perspective, the presence of genera such as *Alternaria*, *Fusarium*, and *Penicillium* is particularly relevant due to their potential to produce mycotoxins and their association with postharvest spoilage. The coexistence of these fungi with the microbial loads described in Table 2 reinforces the need for effective control strategies during both pre- and postharvest stages.

Overall, the results demonstrate that fungal community composition varies notably depending on the vegetable type and cultivar, with cauliflower and Tritón showing higher diversity and abundance of potentially spoilage-related fungi, while Parthenon exhibited a more limited and less diverse fungal profile. These findings support the potential influence of plant-specific factors, including phytochemical composition, on fungal colonization patterns and highlight the importance of considering cultivar selection in strategies aimed at reducing microbial contamination.

### 3.2. Bioactive Compound Characterization

The extraction yield and the content of total phenolic compounds (TPC) and flavonoids obtained from cauliflower and broccoli by-products are presented in Table 4, revealing marked differences depending on both the plant fraction and the cultivar.

Regarding extraction yield, relatively high values were obtained for all samples, ranging from 12.65% to 24.46%. In general, broccoli samples, particularly the Tritón cultivar, showed higher extraction yields, with the highest value observed in Tritón heads (24.46%), followed by Tritón stems (22.77%) and leaves (21.08%). In contrast, cauliflower exhibited lower yields, especially in heads (12.65%). These differences may be attributed to variations in tissue composition, including water content, cell wall structure, and the abundance of extractable metabolites.

In terms of total phenolic content (TPC), significant differences were observed among samples. Broccoli leaves exhibited the highest values, particularly in the Tritón cultivar (179.67 mg GAE/100 g), followed by Parthenon leaves (133.99 mg GAE/100 g), both significantly higher than cauliflower samples. In cauliflower, leaves also showed the highest TPC (86.95 mg GAE/100 g), although values were notably lower than those observed in broccoli. Conversely, stems and heads generally presented lower phenolic contents across all samples. These results suggest that leaves are the primary reservoir of phenolic compounds, likely due to their greater exposure to environmental stressors such as UV radiation, which stimulates the synthesis of protective secondary metabolites.

A similar trend was observed for flavonoid content, with broccoli leaves, particularly Tritón, showing the highest concentration (325.48 mg/L), followed by Parthenon leaves (216.90 mg/L). These values were substantially higher than those found in cauliflower fractions, where flavonoid content ranged between 49.53 and 75.24 mg/L. Notably, Tritón leaves showed significantly higher flavonoid levels compared to all other samples, indicating a strong cultivar-dependent effect on phytochemical accumulation.

Overall, broccoli by-products, especially leaves, demonstrated a significantly richer phenolic and flavonoid profile compared to cauliflower. Additionally, clear differences between broccoli cultivars were observed, with Tritón consistently showing higher concentrations of bioactive compounds than Parthenon. This variability may be attributed to genetic factors, as well as potential differences in physiological responses to environmental conditions.

From a functional perspective, the high levels of phenolic compounds and flavonoids observed in broccoli by-products are particularly relevant, given their well-documented antioxidant and antimicrobial properties. These findings are consistent with the lower microbial loads observed in broccoli samples (Table 2), supporting the hypothesis that the presence of bioactive compounds may contribute to limiting microbial growth.

In conclusion, the results highlight the strong potential of broccoli by-products, especially leaves from the Tritón cultivar, as a valuable source of phenolic compounds and flavonoids. This reinforces their suitability for use in functional applications, including natural antimicrobial formulations and sustainable strategies for food preservation.

The antioxidant activity of cauliflower and broccoli by-products, evaluated using DPPH and ABTS assays, is presented in Table 5. The results revealed notable differences depending on both the plant fraction and the cultivar.

In the DPPH assay, cauliflower stems exhibited the highest antioxidant activity (67.38 mg Trolox/100 g DW), followed by Tritón leaves (49.87 mg Trolox/100 g DW) and Tritón stems (39.39 mg Trolox/100 g DW). In contrast, the lowest values were observed in broccoli stems and heads from the Parthenon cultivar (8.63 and 9.34 mg Trolox/100 g DW, respectively), as well as in Tritón heads (9.03 mg Trolox/100 g DW). These results highlight a strong influence of plant fraction, with stems and leaves generally showing higher radical scavenging capacity than heads.

For the ABTS assay, a different but partially consistent pattern was observed. The highest antioxidant activity was recorded in cauliflower stems (49.53 mg Trolox/100 g DW) and Tritón leaves (41.93 mg Trolox/100 g DW), followed by cauliflower leaves (27.42 mg Trolox/100 g DW) and Parthenon leaves (25.05 mg Trolox/100 g DW). The lowest values were again detected in broccoli stems and heads from the Parthenon cultivar, confirming their lower antioxidant potential. Compared to previous data, ABTS values were more moderate and homogeneous across samples, suggesting a more balanced detection of antioxidant compounds.

Differences between DPPH and ABTS results are commonly reported and can be attributed to their distinct reaction mechanisms. While DPPH primarily detects hydrophobic radical scavengers, ABTS is able to quantify both hydrophilic and lipophilic antioxidants, which may explain the observed variations among samples and fractions.

When these results are compared with the phytochemical data (Table 4), it is evident that antioxidant activity does not strictly correlate with total phenolic and flavonoid contents. Notably, Tritón leaves, although characterized by the highest phenolic content, did not exhibit proportionally higher antioxidant activity in all cases. This may be associated with differences in the qualitative composition of phenolic compounds. In particular, the predominance of highly glycosylated flavonoids in Tritón could be explained by a reduced radical scavenging efficiency compared to less substituted or more reactive phenolic structures present in other samples [27].

Overall, the results confirm that both cauliflower and broccoli by-products possess relevant antioxidant activity, with stems and leaves representing the most active fractions. However, the variability observed between cultivars and analytical methods may be associated with differences in the composition and nature of bioactive compounds, suggesting a possible role of these factors in determining antioxidant potential.

The bioactive compounds identified in cauliflower and broccoli by-products by HPLC- ESI-MS/MS are summarized in Table 6, while their relative abundance across different plant fractions and cultivars is presented in Table 7. Overall, a diverse range of compounds was detected, including phenolic compounds, glucosinolates, fatty acids and their derivatives, as well as other minor constituents.

Among phenolic compounds, several kaempferol derivatives were identified, including glycosylated and acylated forms, along with phenolic acids such as caffeoyl-quinic acid, ferulic acid, protocatechuic acid, and *p*-coumaric acid. These compounds are widely recognized for their antioxidant properties and contribute significantly to the functional value of *Brassica* vegetables.

A clear distinction was observed between cauliflower and broccoli samples (Table 7). Cauliflower showed a more limited phenolic profile, with the presence of simple phenolic acids, particularly protocatechuic acid (peak 8), which was highly abundant in stems. In contrast, broccoli samples, especially the Tritón cultivar, exhibited a much richer and more complex phenolic composition, dominated by kaempferol glycosides (peaks 1, 3, 4, and 6). Notably. Tritón leaves showed exceptionally high levels of these compounds, particularly peak 1 (Km-3-diglucoside-7-glucoside), indicating a strong accumulation of flavonoid derivatives in this fraction.

Glucosinolates were also widely detected, with glucoraphanin and glucobrassicin derivatives being the most representative compounds. These metabolites were more abundant in broccoli than in cauliflower, particularly in the inflorescences (heads), where very high concentrations were observed (e.g., peak 12 in Parthenon heads). This distribution is consistent with the known role of glucosinolates as defense compounds, which tend to accumulate in reproductive tissues to protect against herbivores and pathogens [9,14].

Regarding fatty acids and their derivatives, both cauliflower and broccoli samples showed the presence of compounds such as linolenic and palmitic acids. although their distribution varied among plant fractions. Cauliflower samples generally exhibited higher levels of certain fatty acid derivatives (e.g., peaks 19 and 20), particularly in stems and heads, which may contribute to membrane-related physiological processes and potentially influence oxidative stability.

The analysis of compound distribution (Table 7) revealed a strong influence of both plant fraction and cultivar. Leaves, particularly from the Tritón cultivar, were the richest fraction in phenolic compounds, which is consistent with the high total phenolic and flavonoid contents previously reported (Table 4). However, despite this higher phenolic content, Tritón did not exhibit the highest antioxidant activity in the DPPH assay (Table 5), although it showed the second highest values, particularly in leaves. This discrepancy reinforces the idea that antioxidant capacity depends not only on the total amount of phenolic compounds but also on their qualitative composition. In this context, the predominance of highly glycosylated kaempferol derivatives in Tritón may reduce their radical scavenging efficiency compared to less substituted or more reactive phenolic compounds present in other samples.

In addition, the presence of glucosinolates in high concentrations, particularly in broccoli heads, may also contribute differently to antioxidant and antimicrobial activities, depending on their hydrolysis into biologically active compounds such as isothiocyanates.

Overall, these results demonstrate that broccoli by-products, especially from the Tritón cultivar, represent a rich source of structurally diverse bioactive compounds. The marked differences observed between cultivars and plant fractions highlight the importance of both genetic and anatomical factors in determining phytochemical composition. Furthermore, the combination of phenolic compounds and glucosinolates reinforces the potential of these by-products for functional applications, particularly in the development of natural antioxidant and antimicrobial agents.

The antibacterial activity of cauliflower and broccoli by-product extracts against *Bacillus cereus*, *Staphylococcus aureus*, *Listeria innocua*, and *Listeria monocytogenes* is presented in Table 8. Overall, the results demonstrated a strong dose-dependent inhibitory effect, with higher extract concentrations (80 ppm) generally achieving near-complete inhibition across most samples and bacterial strains. It should be noted that antimicrobial activity was assessed based on optical density measurements, which provide an indication of growth inhibition but do not allow differentiation between bacteriostatic and bactericidal effects.

At 80 ppm, almost all extracts exhibited inhibition values close to or equal to 100%, indicating a potent antibacterial effect regardless of the plant fraction or vegetable type. However, differences became more pronounced at lower concentrations (60 and 40 ppm), allowing a clearer distinction between samples.

Cauliflower extracts showed consistently high antibacterial activity across all fractions and concentrations. Even at 40 ppm, inhibition levels remained relatively high, particularly in stems and heads, suggesting a stable and effective antimicrobial profile. This behavior may be associated with the presence of specific bioactive compounds such as phenolic acids (e.g., protocatechuic and ferulic acids) and glucosinolate derivatives identified in Table 6 and Table 7, which are known to exert antimicrobial effects through membrane disruption and interference with cellular metabolism.

In contrast, broccoli extracts exhibited greater variability depending on cultivar and plant fraction. Parthenon samples generally showed strong antibacterial activity, particularly in stems and heads, which maintained high inhibition levels even at 40 ppm (e.g., up to 92.99% for *B. cereus* and 97.21% for *L. monocytogenes* in heads). Leaves, however, showed a marked decrease in activity at lower concentrations, indicating a lower efficacy compared to other fractions.

Tritón extracts displayed the highest variability among samples. While stems and heads demonstrated strong antibacterial activity at higher concentrations, leaves showed significantly lower inhibition, particularly against *B. cereus* and *S. aureus*, with values dropping to 0–12% at 40 ppm. This result is particularly noteworthy, as Tritón leaves exhibited the highest total phenolic and flavonoid contents (Table 4) yet did not correspondingly show the strongest antibacterial activity.

This apparent discrepancy reinforces the hypothesis that antimicrobial activity is not solely dependent on the total concentration of phenolic compounds, but rather on their specific composition and bioavailability. As shown in Table 6 and Table 7, Tritón samples are rich in highly glycosylated kaempferol derivatives, which may exhibit lower antimicrobial activity compared to simpler phenolic structures or to glucosinolate hydrolysis products such as isothiocyanates, known for their strong bactericidal properties.

Among the tested microorganisms, *B. cereus* and *S. aureus* were generally more susceptible to the extracts, whereas *Listeria monocytogenes* showed more variable responses, particularly at lower concentrations. Nevertheless, several extracts, especially those from Parthenon heads and cauliflower fractions, maintained high inhibitory activity even at 40 ppm, highlighting their potential effectiveness against relevant foodborne pathogens.

Overall, these results suggest that *Brassica* by-products may represent a potential source of natural antibacterial agents. However, the observed differences between cultivars and plant fractions may be associated with variations in phytochemical composition rather than total content, suggesting a possible role of these factors in determining antimicrobial activity. In addition, the results indicate that further optimization of extraction and application conditions may be required to better understand and potentially enhance antimicrobial efficacy.

The antifungal activity of cauliflower and broccoli by-product extracts against *Alternaria alternata*, *Penicillium purpurogenum*, and *Botrytis cinerea* is presented in Table 9. The results revealed a highly variable response depending on the fungal species, extract concentration, plant fraction, and cultivar.

Overall, *Alternaria alternata* was the most susceptible microorganism to the tested extracts. Significant reductions in growth rate and measurable inhibition percentages were observed, particularly in broccoli samples. The highest inhibition was recorded in Parthenon heads at 1250 ppm (37.20%), followed by Tritón stems at 1250 ppm (31.20%), indicating a clear antifungal effect even at lower concentrations. In contrast, cauliflower extracts showed more limited activity, with only moderate inhibition observed in stems (15.20%) and heads (13.20%). These results suggest that broccoli by-products possess greater antifungal potential against *A. alternata*, likely due to their higher content of bioactive compounds such as glucosinolates and phenolic derivatives.

In contrast, *Penicillium purpurogenum* showed complete resistance to all tested extracts. No inhibition was observed at either concentration (0% in all cases), and growth rates remained comparable to control values. This indicates that the bioactive compounds present in the extracts are not effective against this species under the tested conditions, highlighting the species-specific nature of antifungal activity.

Similarly, *Botrytis cinerea* exhibited a high level of resistance, with no measurable inhibition observed in any treatment. In some cases, slightly higher growth rates were recorded compared to the control, suggesting that the extracts did not exert inhibitory effects and may even have provided additional nutrients or favorable conditions for fungal growth. This lack of activity is particularly relevant given the importance of *B. cinerea* as a major postharvest pathogen.

The analysis of growth rates further supports these observations. While statistically significant differences were detected for *A. alternata* (*p* < 0.001), indicating a clear treatment effect, no significant inhibition patterns were observed for *P. purpurogenum*, and only limited differences were detected for *B. cinerea* (*p* = 0.008), likely reflecting minor variations rather than a true antifungal effect.

Differences between cultivars were also evident. Parthenon extracts, particularly from heads, showed the highest antifungal activity against *A. alternata*, which is consistent with their relatively strong antibacterial performance (Table 8). Tritón extracts, despite having higher phenolic content (Table 4), did not consistently show higher antifungal activity, again reinforcing the importance of compound composition over total content. As previously discussed, the predominance of glycosylated flavonoids in Tritón may reduce their biological activity compared to more reactive compounds such as isothiocyanates derived from glucosinolates.

Overall, the results indicate that the antifungal activity of *Brassica* by-product extracts may be associated with the target microorganism. While a moderate inhibitory effect was observed against *A. alternata*, no significant activity was detected against *P. purpurogenum* or *B. cinerea*. These findings suggest that these extracts may have potential as selective antifungal agents; however, their application in postharvest disease control could be explained by the need for further optimization, including adjustments in concentration, combination with other treatments, or improved availability of active compounds.

### 3.3. Multivariate Analysis

**Figure 1:**
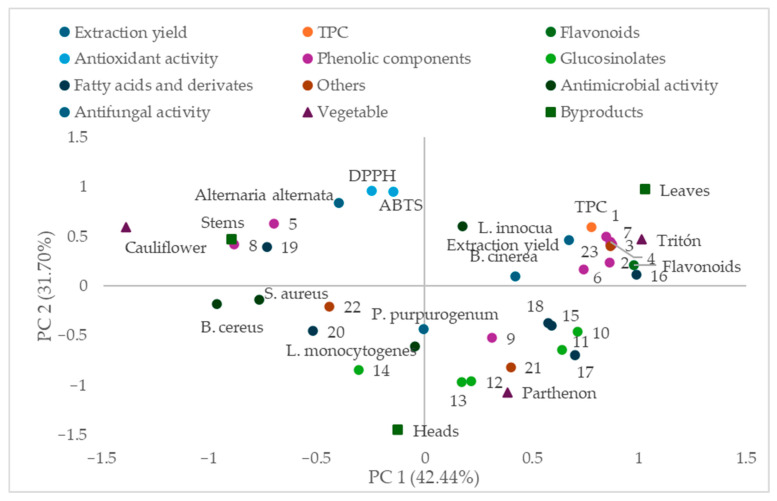
Principal Component Analysis (PCA) projection on the plane defined by PC1 (42.44%) and PC2 (31.70%) showing the combined distribution of the analyzed variables and samples. The variables include phenolic compounds (**1**–**9**), glucosinolate (**9**–**14**), fatty acids and derivatives (**15**–**20**), others (**21**–**23**), total phenolic compounds (TPC), antioxidant activity (DPPH and ABTS), and antimicrobial activity against *L. innocua, L. monocytogenes, B. cereus, and S. aureus* and antifungal against *Alternaria alternata, P. purpurogenum* and *B. cinerea*. The distribution of the samples according to vegetable (Cauliflower, Tritón and Parthenon) and by-products (leaves, heads and stems). The numerical code for compounds corresponds to those identified in Table 6.

Figure 1 presents the principal component analysis (PCA), which was selected as a multivariate approach to explore and visualize the relationships among phytochemical composition and biological activity variables, as well as to reduce data dimensionality. The projection of samples and variables onto the plane defined by principal component 1 (PC1) and principal component 2 (PC2), which explain 42.44% and 31.70% of the total variance, respectively. PC1 is mainly associated with the presence of phenolic compounds and flavonoids, as well as total phenolic content (TPC), which are positioned on one side of the plot and are closely related to samples from broccoli leaves, particularly from the Tritón cultivar, indicating a higher concentration of these compounds in this fraction.

Moreover, several phenolic compounds, including kaempferol derivatives (peaks 1, 3, 4, and 6) and other identified phenolics, are located near the same region, suggesting their contribution to the phytochemical richness of Tritón samples. In contrast, antioxidant activity measured by DPPH and ABTS appears distributed differently, showing a closer association with Parthenon samples, which supports the previously observed discrepancy between total phenolic content and antioxidant capacity.

PC2 differentiates samples mainly according to their antimicrobial activity, including antibacterial effects against *Listeria monocytogenes*, *Listeria innocua*, *Bacillus cereus*, and *Staphylococcus aureus*, as well as antifungal activity against *Alternaria alternata*. Parthenon samples, particularly stems and heads, are positioned closer to these variables, indicating a stronger relationship with biological activity despite their lower total phenolic content.

At the opposite side of the plot, variables related to fatty acids and other minor compounds are grouped, showing a weaker association with biological activities. Additionally, antifungal activity against *Penicillium purpurogenum* and *Botrytis cinerea* does not show a clear clustering pattern, which is consistent with the limited inhibitory effects observed for these microorganisms.

The distribution of the samples according to vegetable type and plant fraction highlights the strong influence of both cultivar and anatomical part on phytochemical composition and functional properties. Broccoli leaves are clearly separated from stems and heads, reinforcing their role as the most bioactive fraction.

In summary, this PCA provides a comprehensive visualization of the relationships between phytochemical composition and biological activities, suggesting patterns that are consistent with the trends observed throughout the study. The results may be associated with differences in compound composition rather than total content, suggesting a possible role of these factors in determining functional properties.

## 4. Discussion

The present study provides an integrated evaluation of broccoli and cauliflower by-products, combining microbial characterization, fungal identification, phytochemical profiling, antioxidant capacity, and antimicrobial activity, highlighting their potential as functional and sustainable resources.

Microbiological analysis revealed that microbial loads were significantly influenced by plant fraction and vegetable type, with leaves consistently showing higher contamination levels. This observation is consistent with previous studies reporting that aerial plant tissues are more exposed to environmental contamination sources such as soil particles, irrigation water, and airborne microorganisms [10,28,29,30]. In contrast, broccoli samples, particularly the Parthenon cultivar, exhibited lower microbial counts and reduced prevalence of pathogens such as *Listeria monocytogenes*, suggesting that intrinsic plant characteristics may contribute to limiting microbial colonization. This behavior may be associated not only with differences in exposure but also with intrinsic plant traits, including tissue architecture and phytochemical composition. Broccoli tissues are known to contain higher concentrations of glucosinolates and phenolic compounds, which may contribute to limiting microbial establishment and proliferation, whereas the more compact structure and moisture-retention capacity of cauliflower curds could favor microbial persistence.

The molecular identification of fungal isolates confirmed the predominance of genera commonly associated with plant materials and postharvest environments, including *Alternaria*, *Penicillium*, and *Fusarium*. These genera are frequently reported as major contributors to spoilage and mycotoxin production in fresh produce [31,32,33]. Furthermore, fungal alterations caused by these molds have been reported as the main cause of losses in broccoli crops in recent years [34,35,36,37]. The higher diversity observed in cauliflower and Tritón samples is consistent with their greater microbial loads and may reflect more favorable ecological conditions for fungal colonization. In contrast, the reduced diversity detected in the Parthenon cultivar suggests a more restrictive microbial environment, potentially linked to cultivar-specific metabolic profiles and the presence of bioactive compounds capable of modulating fungal growth and community structure.

The extraction method used for obtaining the plant extracts in this study was selected due to its suitability for analytical purposes, enabling the efficient recovery and characterization of glucosinolates and phenolic compounds. The observed extraction performance reflects inherent differences in plant matrix composition, including structural characteristics and the availability of extractable metabolites across samples. This type of extraction approach is widely applied in phytochemical studies for the compositional analysis of *Brassica* matrices [18], although it is not intended to represent an optimized process for industrial-scale production. Therefore, the results should be interpreted within the context of compositional profiling and comparative evaluation rather than extraction efficiency. Further research is required to assess compound-specific yields, as well as the techno-economic feasibility, environmental impact, and scalability of these extraction processes under industrial conditions.

Phytochemical characterization revealed a diverse profile of bioactive compounds, including phenolic acids, flavonoids (notably kaempferol derivatives), and glucosinolates such as glucoraphanin and glucobrassicin. Broccoli by-products, especially leaves, exhibited a richer and more complex composition compared to cauliflower, in agreement with previous studies on *Brassica* vegetables [38,39,40,41]. However, clear differences between cultivars were observed, with Tritón showing higher total phenolic and flavonoid contents, while Parthenon appeared to present a more balanced or potentially more bioactive profile. Other studies also highlight the influence of cultivar on the bioactive compound content of broccoli [5,42].

These compositional differences were reflected in the antioxidant activity assays, suggesting that activity may be associated with extract composition and potential interactions between compounds rather than total phenolic content. Although Tritón samples, particularly leaves, showed high antioxidant capacity and ranked among the most active extracts, they did not consistently exhibit the highest radical scavenging activity. In the DPPH assay, cauliflower stems showed the highest activity, while Tritón leaves presented the second highest values. This result may be explained by the specific phenolic profile of cauliflower stems, which are characterized by a higher proportion of simple phenolic acids, such as protocatechuic and ferulic acids, known for their strong hydrogen-donating ability and high reactivity in DPPH assays. In contrast, Tritón leaves, despite their higher total phenolic content, are dominated by glycosylated flavonoids, particularly kaempferol derivatives, whose antioxidant efficiency may be reduced due to steric hindrance and lower accessibility of hydroxyl groups. This lack of direct correlation between total phenolic content and antioxidant activity has been widely reported and is attributed to differences in phenolic structure, including glycosylation patterns, which can reduce radical scavenging efficiency [43,44,45]. In addition, other compounds such as glucosinolates and their degradation products may contribute differently depending on the assay conditions [46,47]. The pigments and vitamins naturally present in broccoli also contribute significantly to antioxidant activity [48,49]; however, their effect may be less pronounced in DPPH assays, which are more sensitive to specific types of radical scavengers.

The antibacterial activity results demonstrated a strong inhibitory effect of all extracts at higher concentrations, with particular effectiveness against *Bacillus cereus*, *Staphylococcus aureus*, and *Listeria* spp. At lower concentrations, Parthenon extracts, especially from stems and heads, maintained higher activity compared to Tritón, despite the latter having higher phenolic content. This finding reinforces the importance of phytochemical composition over total content. Glucosinolate hydrolysis products, such as sulforaphane, are known to exhibit strong antimicrobial activity through membrane disruption and interference with cellular metabolism [7,50], which may explain the higher efficacy observed in certain samples. In some cultivars, stem and inflorescence antimicrobial effects against *B. cereus*, *S. aureus*, and *Listeria innocua* correlate with higher levels of fatty-acid derivatives [5].

In contrast, antifungal activity was more selective. Moderate inhibition was observed against *Alternaria alternata*, whereas *Penicillium purpurogenum* and *Botrytis cinerea* showed high resistance to all extracts. This species-specific response is consistent with previous studies and reflects differences in fungal physiology, cell wall composition, and detoxification mechanisms [51,52]. In this context, it has been reported that crude extracts from broccoli florets and stems effectively inhibit *Colletotrichum gloeosporioides* and *Aspergillus niger* and that protease treatment reduces antifungal activity by approximately 60%, indicating the involvement of heat-stable proteinaceous peptides [8]. Together with evidence showing that glucosinolates from leaf extracts exert strong in vitro antifungal effects against phytopathogenic fungi such as *Alternaria*, *Sclerotinia*, and *Rhizoctonia*, particularly when their hydrolysis is promoted by myrosinase activity [15], these results suggest that *Brassica* by-product extracts may be more suitable for targeted, mechanism-specific antifungal applications rather than broad-spectrum use, while also indicating that their effectiveness may require further optimization or fractionation to enhance activity against less susceptible species.

Overall, the results demonstrate a clear relationship between phytochemical composition and biological activity, as well as the importance of plant fraction and cultivar selection. Leaves, particularly from broccoli, represent the most promising fraction due to their high content of bioactive compounds. Moreover, Tritón stands out for its high phenolic content and antioxidant capacity, while Parthenon exhibits a more consistent antimicrobial performance.

From an applied perspective, these findings support the valorization of broccoli by-products as sustainable sources of natural antioxidants and antimicrobials. Their incorporation into food preservation systems and postharvest treatments could contribute to reducing microbial contamination and extending shelf life, in line with current trends toward sustainable and circular food systems [53,54]. However, the variability observed among cultivars and target microorganisms highlights the need for further optimization of extraction methods and application strategies.

## 5. Conclusions

Broccoli by-products, particularly leaves, may represent a potential source of bioactive compounds with antioxidant and antimicrobial properties of interest for food-related applications. Microbial characterization indicated relatively low contamination levels in certain cultivars, suggesting a possible relationship between phytochemical composition and microbial behavior. Biological activity appeared to be more closely associated with compound composition than with total phenolic content, pointing to a potential role of specific phenolic structures and glucosinolate derivatives in determining functional effects. Extracts showed inhibitory activity against relevant foodborne pathogens under in vitro tested conditions, while antifungal effects were more limited and species-dependent.

These findings suggest that broccoli by-products may have potential as natural ingredients for food preservation and postharvest management, contributing to more sustainable food systems. However, their practical application remains dependent on factors such as cultivar, plant fraction, target microorganism, and validation under real food-system conditions. Further studies are required to identify the key active compounds, confirm their effectiveness in real food matrices, and evaluate industrial feasibility, scalability, and techno-economic applicability.

## Figures and Tables

**Table 1:** Culture media and incubation conditions used for microbial enumeration.

Agar	ID	Specific Microorganism	Incubation Temperature	Incubation Time
Plate Count Agar	PCA	Mesophilic aerobic bacteria	30 °C	24 h
Man–Rogosa–Sharpe Agar	MRS	*Lactobacillus* spp.	30 °C	48 h
Baird Parker Agar	BP	*Staphylococcus* spp.	37 °C	48 h
Mannitol Salt Agar	MSA	Micrococcaceae	37 °C	48 h
Violet Red Bile Glucose Agar	VRBG	Enterobacteriaceae	30 °C	24 h
Tryptone Bile X-Glucuronide Agar	TBX	*Escherichia coli*	37 °C	24 h
Xylose Lysine Deoxycholate Agar	XLD	*Salmonella* sp.	37 °C	48 h
Listeria Agar	LA	*Listeria monocytogenes*	37 °C	48 h
Potato Dextrose Agar	PDA	Yeast and Molds	25 °C	48 h

**Table 2:** Microbial counts (log_10_ CFU/g) in different fractions of cauliflower and broccoli samples.

		Molds	Yeast	Mesophilic Aerobic Bacteria	Enterobacteriaceae
**Vegetable**	**Byproduct**	Mean SD *	**Mean SD**	**Mean SD**	**Mean SD**
Cauliflower	Leaves	4.86 ^a^ ± 0.26	2.00 ^ab^ ± 0.00	6.99 ^a^ ± 0.01	6.85 ^a^ ± 0.00
	Stems	5.42 ^a^ ± 0.10	1.00 ^ab^ ± 0.18	6.90 ^a^ ±0.05	6.30 ^a^ ± 0.25
	Heads	3.70 ^b^ ± 0.02	4.78 ^a^ ± 0.10	6.76 ^ab^ ±0.06	6.11 ^a^ ± 0.01
Parthenon	Leaves	2.74 ^c^ ± 0.04	2.15 ^ab^ ± 0.15	6.63 ^ab^ ±0.01	6.06 ^a^ ± 0.02
	Stems	2.00 ^d^ ± 0.00	1.00 ^ab^ ± 0.10	5.05 ^d^ ±0.02	4.80 ^b^ ± 0.24
	Heads	0.00 ^e^ ± 0.00	0.00 ^b^ ± 0.00	5.28 ^d^ ±0.02	4.30 ^bc^ ± 0.30
Tritón	Leaves	2.69 ^c^ ± 0.09	0.00 ^b^ ± 0.00	5.31 ^cd^ ± 0.00	3.94 ^bc^ ± 0.00
	Stems	2.30 ^cd^ ± 0.00	0.00 ^b^ ± 0.00	4.91 ^d^ ± 0.41	3.65 ^c^ ± 0.00
	Heads	2.15 ^cd^ ±0.15	0.00 ^b^ ± 0.00	6.06 ^bc^ ± 0.02	4.70 ^b^ ± 0.00
		***Staphylococcus aureus***	***Staphylococcus*** ** sp.**	**Micrococcaceae**	***L. monocytogenes***
**Vegetable**	**Byproduct**	Mean SD *	**Mean SD**	**Mean SD**	**Mean SD**
Cauliflower	Leaves	2.24 ^ab^ ± 0.24	3.50 ^ab^ ± 0.05	3.19 ^a^ ± 0.04	2.15 ^c^ ± 0.15
	Stems	3.50 ^a^ ± 0.50	4.55 ^a^ ± 0.17	1.00 ^b^ ± 0.02	2.82 ^b^ ± 0.22
	Heads	1.00 ^ab^ ± 0.00	0.00 ^d^ ± 0.00	4.10 ^a^ ± 0.02	0.00 ^d^ ± 0.00
Parthenon	Leaves	2.24 ^ab^ ± 0.24	2.30 ^abc^ ± 0.30	3.22 ^a^ ± 0.04	0.00 ^d^ ± 0.00
	Stems	1.00 ^ab^ ± 0.00	2.00 ^bcd^ ± 0.00	2.24 ^ab^ ± 0.24	0.00 ^d^ ± 0.00
	Heads	0.00 ^b^ ± 0.00	2.00 ^bcd^ ± 0.00	2.24 ^ab^ ± 0.24	0.00 ^d^ ± 0.00
Tritón	Leaves	2.94 ^a^ ± 0.10	3.66 ^ab^ ± 0.08	4.10 ^a^ ± 0.20	3.59 ^a^ ± 0.15
	Stems	0.00 ^b^ ± 0.00	1.15 ^cd^ ± 0.15	3.15 ^a^ ± 0.03	2.00 ^c^ ± 0.00
	Heads	0.00 ^b^ ± 0.00	2.39 ^abc^ ± 0.09	3.30 ^a^ ± 0.07	2.85 ^b^ ± 0.00

* SD: standard deviation. Superscript (a,b,c,d): mean values with different letters indicate significant differences (*p* < 0.05) between samples by columns.

**Table 3:** Molecular identification of fungal isolates from cauliflower and broccoli by-products based on ITS sequencing.

Vegetable	Isolate	Species Identified	Identity (%)	Accession
Cauliflower	C2	*Alternaria alternata*	100%	PV550434.1
	C4	*Penicillium purpurogenum*	100%	MN086355.1
	C5	*Alternaria alternata*	100%	PV550434.1
	C6	*Penicillium variabile*	100%	MW856768.1
Parthenon	P2	*Fusarium oxysporum*	100%	MT560381.1
	P5	*Penicillium dimorphosporum*	98%	MH859796.1
	P8	*Penicillium purpurogenum*	100%	MN086355.1
Tritón	T1	*Penicillium chalabudae*	100%	NR_144845.1
	T2	*Penicillium chalabudae*	99%	NR_144845.1
	T3	*Penicillium menonorum*	99%	OP179069.1
	T4	*Scolecobasidium ramosum*	94%	NR_155606.1
	T5	*Alternaria alternata*	100%	PV550434.1
	T6	*Fusarium oxysporum*	100%	MT560381.1
	T7	*Penicillium pimiteouiense*	100%	MW287274.1

**Table 4:** Extraction yield (%), total phenolic content (mg GAE/100 g DW), and flavonoid content (mg/L) of cauliflower and broccoli by-products.

		Extraction Yield	TPC	Flavonoids
Vegetable	Byproduct	Mean SD *	Mean SD	Mean SD
Cauliflower	Leaves	20.84 ^ab^ ± 2.71	44.94 ^de^ ± 7.65	52.96 ^d^ ± 12.34
	Stems	16.66 ^bc^ ± 1.29	86.95 ^c^ ± 4.01	75.24 ^cd^ ± 15.82
	Heads	12.65 ^c^ ± 2.46	25.18 ^e^ ± 0.72	49.53 ^d^ ± 10.37
Parthenon	Leaves	18.11 ^b^ ± 1.59	133.99 ^b^ ± 16.56	216.90 ^b^ ± 30.13
	Stems	16.57 ^bc^ ± 1.56	27.43 ^e^ ± 1.27	80.67 ^cd^ ± 7.20
	Heads	16.12 ^bc^ ± 1.79	39.01 ^de^ ± 4.56	118.02 ^cd^ ± 10.02
Tritón	Leaves	21.08 ^a^ ± 1.32	179.70 ^a^ ± 5.18	325.48 ^a^ ± 44.47
	Stems	22.77 ^a^ ± 2.18	48.70 ^de^ ± 7.97	88.08 ^cd^ ± 14.53
	Heads	24.46 ^a^ ± 2.20	55.98 ^d^ ± 2.62	139.46 ^c^ ± 16.84

* SD: standard deviation. Superscript (a,b,c,d,e): mean values with different letters indicate significant differences (*p* < 0.05) between samples.

**Table 5:** Antioxidant activity determined by DPPH and ABTS (mg Trolox per 100 g dry weight) assays for extracts obtained from cauliflower and broccoli by-products.

		DPPH	ABTS
Vegetable	Byproduct	Mean SD *	Mean SD
Cauliflower	Leaves	31.66 ^cd^ ± 3.04	27.42 ^b^ ± 9.13
	Stems	67.38 ^a^ ± 10.03	49.53 ^a^ ± 3.37
	Heads	16.01 ^ef^ ± 1.23	16.68 ^cde^ ± 2.15
Parthenon	Leaves	23.01 ^de^ ± 2.65	25.05 ^bc^ ± 1.03
	Stems	8.63 ^f^ ± 0.94	7.19 ^e^ ± 0.63
	Heads	9.34 ^f^ ± 0.46	12.32 ^de^ ± 0.63
Tritón	Leaves	49.87 ^b^ ± 1.62	41.93 ^a^ ± 1.53
	Stems	39.39 ^bc^ ± 3.04	17.38 ^cd^ ± 0.09
	Heads	9.03 ^f^ ± 3.35	11.32 ^de^ ± 0.30

* SD: standard deviation. Superscript (a,b,c,d,e,f): mean values with different letters indicate significant differences (*p* < 0.05) between samples.

**Table 6:** Compounds identified in cauliflower and broccoli by-product extracts analyzed by HPLC-ESI-QTOF.

Peak	Rt (min)	[M − H]^−^	HPLC-ESI(-)-MSn Experiment *m*/*z*	Compounds Identified
Phenolic components				
1	12.26	**771**	609; 610	Km-3-diglucoside-7-glucoside ^a^
2	12.43	**353**	135; 179; 191	Caffeoyl-quinic acid ^b^
3	12.54	**933**	771; 772	Km-3-diglucoside-7-diglucoside ^a^
4	12.78	**947**	785; 786	Km-3-feruloyldiglucoside-7-glucoside ^a^
5	14.37	**193**	149	Ferulic acid ^b^
6	13.77	**1109**	785; 786	Km-3-O-feruloyldiglucoside-7-O-diglucoside ^b^
7	15.25	**285**	571	Kaempferol ^c^
8	21.67	**153**	-	Protocatechuic acid ^d^
9	24.87	**163**	164	p-coumaric acid ^b^
Glucosinolates				
10	3.14	**436**	437	Glucoraphanin isomer 1 ^a^
11	3.97	**436**	437	Glucoraphanin isomer 2 ^a^
12	12.75	**447**	448	Glucobrassicin ^a^
13	13.15	**477**	478	Methoxyglucobrassicin 1 ^a^
14	13.62	**477**	446; 447; 478	Methoxyglucobrassicin 2 ^a^
Fatty acids and derivates				
15	15.73	**327**	211; 328; 677	FA 18:2 + 3O ^a^
16	16.10	**329**	211; 330	FA 18:1 + 3O ^a^
17	18.25	**255**	256	Palmitic acid ^a^
18	18.93	**277**	224; 278	Linolenic acid ^e^
19	25.82	**277**	278	Linolenic acid isomer ^a^
20	26.04	**255**	256	Palmitic acid isomer ^e^
Others				
21	2.86	**195**	129	Gluconic acid ^a^
22	4.02	**128**	200; 290	Fructosyl-pyroglutamate derivate ^e^
23	13.52	**771**	772; 933	Unknown 1

Values correspond to the *m*/*z* of deprotonated molecular ions [M − H]^−^ acquired in negative electrospray ionization (ESI^−^) mode. Both nominal and exact mass values are reported, depending on the compound and data processing conditions. The most representative fragment ions are highlighted in bold within the MS/MS column. Compound identification was supported by comparison with MassBank and literature data: ^a^ [5]; ^b^ [24]; ^c^ [25]; ^d^ [26]; ^e^ [23].

**Table 7:** Relative abundance (peak area, arbitrary units) of compounds identified in cauliflower and broccoli by-product extracts across different plant fractions and cultivars.

	Cauliflower			Parthenon			Tritón		
Peak	Leaves	Stems	Heads	Leaves	Stems	Heads	Leaves	Stems	Heads
Phenolic components									
1	nd *	nd	nd	194.34	5.28	18.19	1320.88	nd	63.54
2	nd	nd	nd	106.41	27.45	110.01	587.63	174.05	266.53
3	nd	nd	nd	198.32	nd	nd	328.99	nd	nd
4	nd	nd	nd	255.38	nd	nd	537.95	nd	nd
5	15.06	19.09	nd	nd	nd	nd	nd	nd	nd
6	nd	nd	nd	205.78	nd	nd	84.22	nd	nd
7	nd	nd	nd	247.88	nd	nd	451.94	nd	13.89
8	48.40	4183.94	13.67	nd	nd	nd	nd	nd	nd
9	nd	nd	nd	108.21	59.08	75.77	nd	nd	nd
Glucosinolates									
10	nd	nd	nd	11.45	61.00	544.37	453.66	222.94	376.75
11	nd	nd	nd	4.35	11.85	288.15	174.22	59.38	167.80
12	38.84	73.47	79.93	32.99	19.94	859.69	175.84	10.29	154.96
13	85.57	154.15	31.64	32.99	19.94	859.69	175.84	10.29	154.96
14	44.20	67.40	14.63	10.55	42.57	201.47	nd	nd	nd
Fatty acids and derivates									
15	nd	nd	nd	254.30	22.45	160.52	62.77	0.58	3.84
16	nd	nd	nd	45.25	nd	61.82	140.58	15.52	15.36
17	nd	nd	nd	51.77	nd	110.70	38.69	50.70	41.23
18	11.25	nd	154.77	133.87	56.45	52.35	119.84	15.67	30.77
19	180.98	236.67	167.79	63.41	36.74	28.52	57.08	16.34	64.11
20	77.28	nd	336.59	117.10	122.14	37.08	37.90	49.45	34.59
Others									
21	nd	nd	nd	33.69	nd	181.46	17.08	nd	208.16
22	352.60	138.31	146.19	134.20	206.86	173.65	22.33	10.76	200.13
23	nd	nd	nd	97.11	nd	nd	149.36	nd	nd

The numerical code corresponds to the compounds listed in Table 3. * nd—not detected.

**Table 8:** Antibacterial activity of extracts obtained from cauliflower and broccoli by-products against *Bacillus cereus*, *Staphylococcus aureus*, *Listeria innocua*, and *Listeria monocytogenes* (growth inhibition, % vs. untreated control).

		*Bacillus cereus*			*Staphylococcus aureus*			*Listeria innocua*			*Listeria monocytogenes*		
Vegetable	Byproduct	80 ppm	60 ppm	40 ppm	80 ppm	60 ppm	40 ppm	80 ppm	60 ppm	40 ppm	80 ppm	60 ppm	40 ppm
Cauliflower	Leaves	100.00 ^a^	95.98 ^a^	96.94 ^a^	100.00 ^a^	92.77 ^ab^	62.62 ^c^	100.00 ^a^	89.84 ^ab^	77.05 ^bc^	100.00 ^a^	95.74 ^ab^	78.92 ^bc^
	Stems	100.00 ^a^	100.00 ^a^	99.89 ^a^	100.00 ^a^	98.95 ^a^	77.05 ^b^	100.00 ^a^	100.00 ^a^	63.84 ^cd^	100.00 ^a^	92.35 ^bc^	65.45 ^cd^
	Heads	99.92 ^a^	97.91 ^a^	95.46 ^a^	100.00 ^a^	92.59 ^ab^	63.84 ^c^	100.00 ^a^	100.00 ^a^	62.62 ^cd^	100.00 ^a^	100.00 ^a^	77.10 ^c^
Parthenon	Leaves	98.73 ^a^	79.71 ^b^	20.28 ^c^	99.82 ^a^	59.44 ^c^	36.14 ^d^	97.65 ^a^	86.11 ^b^	96.74 ^a^	99.87 ^a^	99.57 ^a^	65.11 ^cd^
	Stems	100.00 ^a^	97.59 ^a^	85.15 ^a^	100.00 ^a^	99.78 ^a^	96.74 ^a^	100.00 ^a^	97.31 ^a^	66.38 ^c^	75.01 ^b^	66.35 ^d^	37.96 ^e^
	Heads	100.00 ^a^	97.26 ^a^	92.99 ^a^	99.45 ^a^	98.80 ^a^	66.38 ^c^	100.00 ^a^	100.00 ^a^	36.14 ^e^	98.46 ^a^	98.02 ^a^	97.21 ^ab^
Tritón	Leaves	44.30 ^b^	12.63 ^c^	0.00 ^c^	47.50 ^b^	26.67 ^d^	12.39 ^e^	63.46 ^b^	52.97 ^c^	93.22 ^ab^	97.53 ^a^	88.42 ^c^	48.05 ^de^
	Stems	100.00 ^a^	99.85 ^a^	99.43 ^a^	97.10 ^a^	96.55 ^ab^	93.22 ^a^	100.00 ^a^	100.00 ^a^	42.66 ^d^	55.27 ^c^	33.15 ^e^	18.83 ^f^
	Heads	100.00 ^a^	100.00 ^a^	50.93 ^b^	98.09 ^a^	80.73 ^b^	42.66 ^d^	100.00 ^a^	95.63 ^ab^	12.39 ^f^	100.00 ^a^	100.00 ^a^	98.62 ^a^

Superscript (a,b,c,d,e,f): mean values with different letters indicate significant differences (*p* < 0.05) between samples.

**Table 9:** Antifungal activity of extracts obtained from cauliflower and broccoli by-products against *Alternaria alternata*, *Penicillium purpurogenum* and *Botrytis cinerea* (growth rate, mm/day, and growth inhibition, % vs. untreated control).

			*Alternaria alternata*		*Penicillium purpurogenum*		*Botrytis cinerea*	
			Growth Rate (mm/day)	Inhibition D7 (%)	Growth Rate (mm/day)	Inhibition D7 (%)	Growth Rate (mm/day)	Inhibition D7 (%)
Vegetable	Byproduct	ppm	Mean SD *	Mean SD	Mean SD	Mean SD	Mean SD	Mean SD
Control			5.00 ^ab^ ± 0.3	-	6.86 ± 0.46	-	8.83 ^b^ ± 0.16	-
Control H_2_O			4.79 ^ab^ ± 0.44	-	6.46 ± 0.08	-	8.81 ^b^ ± 0.24	-
Cauliflower	Leaves	2500	5.16 ^ab^ ± 0.33	0.00 ^b^ ± 0.00	6.23 ± 0.80	0.00 ± 0.00	9.41 ^ab^ ± 0.27	0.00 ± 0.00
		1250	5.60 ^ab^ ± 0.38	0.00 ^b^ ± 0.00	6.03 ± 0.32	0.00 ± 0.00	9.15 ^ab^ ± 0.59	0.00 ± 0.00
	Stems	2500	4.59 ^ab^ ± 0.09	15.20 ^ab^ ± 1.64	7.01 ± 0.27	0.00 ± 0.00	9.62 ^ab^ ± 0.19	0.00 ± 0.00
		1250	6.09 ^a^ ± 1.60	0.00 ^b^ ± 0.00	6.49 ± 0.28	0.00 ± 0.00	9.35 ^ab^ ± 0.35	0.00 ± 0.00
	Heads	2500	4.85 ^ab^ ± 0.15	0.00 ^b^ ± 0.00	6.14 ± 0.46	0.00 ± 0.00	9.42 ^ab^ ± 0.35	0.00 ± 0.00
		1250	4.58 ^ab^ ± 0.26	13.20 ^ab^ ± 5.09	6.47 ± 0.08	0.00 ± 0.00	9.47 ^ab^ ± 0.14	0.00 ± 0.00
Parthenon	Leaves	2500	4.57 ^ab^ ± 0.17	10.80 ^ab^ ± 1.49	7.00 ± 0.35	0.00 ± 0.00	9.79 ^ab^ ± 0.06	0.00 ± 0.00
		1250	4.51 ^ab^ ± 0.12	21.60 ^ab^ ± 1.99	6.95 ± 0.25	0.00 ± 0.00	9.69 ^ab^ ± 0.07	0.00 ± 0.00
	Stems	2500	4.60 ^ab^ ± 0.21	10.80 ^ab^ ± 1.69	7.20 ± 0.49	0.00 ± 0.00	9.50 ^ab^ ± 0.17	0.00 ± 0.00
		1250	4.29 ^b^ ± 0.24	14.40 ^ab^ ± 3.34	7.27 ± 0.26	0.00 ± 0.00	9.48 ^ab^ ± 0.81	0.00 ± 0.00
	Heads	2500	5.31 ^ab^ ± 0.29	13.20 ^ab^ ± 1.87	7.04 ± 0.28	0.00 ± 0.00	9.45 ^ab^ ± 0.26	0.00 ± 0.00
		1250	4.41 ^b^ ± 0.30	37.20 ^a^ ± 5.27	6.93 ± 0.39	0.00 ± 0.00	9.39 ^ab^ ± 0.09	0.00 ± 0.00
Tritón	Leaves	2500	5.18 ^ab^ ± 0.28	10.40 ^ab^ ± 2.71	6.60 ± 0.41	0.00 ± 0.00	9.59 ^ab^ ± 0.23	0.00 ± 0.00
		1250	5.36 ^ab^ ± 0.46	17.60 ^ab^ ± 2.71	6.44 ± 0.40	0.00 ± 0.00	9.50 ^ab^ ± 0.05	0.00 ± 0.00
	Stems	2500	5.15 ^ab^ ± 0.35	12.48 ^ab^ ± 4.07	6.53 ± 0.23	0.00 ± 0.00	9.98 ^a^ ± 0.56	0.00 ± 0.00
		1250	4.47 ^ab^ ± 0.03	31.20 ^ab^ ± 2.36	6.45 ± 0.14	0.00 ± 0.00	9.91 ^a^ ± 0.13	0.00 ± 0.00
	Heads	2500	4.81 ^ab^ ± 0.29	4.80 ^ab^ ± 1.4	7.09 ± 0.9	0.00 ± 0.00	9.58 ^ab^ ± 0.17	0.00 ± 0.00
		1250	4.45 ^ab^ ± 0.28	10.80 ^ab^ ± 1.69	5.95 ± 0.41	0.00 ± 0.00	9.32 ^ab^ ± 0.29	0.00 ± 0.00
*p*-values			<0.001	<0.001	0.071	-	0.008	-

* SD: standard deviation. Superscript (a,b): mean values with different letters indicate significant differences (*p* < 0.05) between samples.

## Data Availability

The original contributions presented in this study are included in the article. Further inquiries can be directed to the corresponding author.
